# The Manipulation of RNA‐Guided Nucleic Acid Cleavage with Ninhydrin Chemistry

**DOI:** 10.1002/advs.201903770

**Published:** 2020-05-26

**Authors:** Shao‐Ru Wang, Hai‐Yan Huang, Jian Liu, Lai Wei, Ling‐Yu Wu, Wei Xiong, Ping Yin, Tian Tian, Xiang Zhou

**Affiliations:** ^1^ College of Chemistry and Molecular Sciences Key Laboratory of Biomedical Polymers of Ministry of Education Wuhan University Wuhan Hubei 430072 China; ^2^ Sauvage Center for Molecular Sciences Wuhan University Wuhan 430072 China; ^3^ Hubei Province Key Laboratory of Allergy and Immunology Wuhan University Wuhan 430071 China; ^4^ National Key Laboratory of Crop Genetic Improvement and National Center of Plant Gene Research Huazhong Agricultural University Wuhan 430070 China

**Keywords:** CRISPR, ninhydrin chemistry, postsynthetic modification

## Abstract

CRISPR (clustered regularly interspaced short palindromic repeats) systems have been established as valuable genome‐editing tools. Controlling CRISPR systems has high biological significance and this field has garnered intense interest. There is a considerable need for simple approaches with no need for protein engineering. The CRISPR systems usually require a guide RNA (gRNA) moiety to recruit and direct the nuclease complexes. In this respect, the ninhydrin (1,2,3‐indantrione monohydrate) seems to have considerable potential, as yet unexploited, for modifying gRNA. In this study, ninhydrin chemistry is explored for reversible postsynthetic modification of gRNA molecules. It is further shown that ninhydrin chemistry is efficient in modulating two important CRISPR systems. Thus, ninhydrin chemistry exhibits potential applications in future chemical biology studies.

## Graphical abstract

In this study, ninhydrin chemistry is explored for reversible modification of guide RNA molecules. It is further shown that ninhydrin chemistry is useful in modulating two important clustered regularly interspaced short palindromic repeats systems. Thus, ninhydrin chemistry exhibits potential applications in future chemical biology studies.

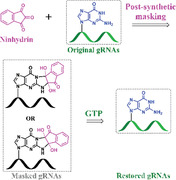

## 1 Introduction

The CRISPR (clustered regularly interspaced short palindromic repeats) system is a prevalent prokaryotic defense system against foreign genetic elements.^[^ ^1^, ^2^, ^3^, ^4^ ^]^ CRISPR systems have been established as valuable genome‐editing tools.^[^ ^5^, ^6^, ^7^ ^]^ The CRISPR system usually requires a guide RNA (gRNA) moiety to recruit and direct the CRISPR‐associated proteins (Cas) ).^[^ ^5^, ^6^, ^7^ ^]^ Controlling CRISPR systems has high biological significance and this field has garnered intense interests.^[^ ^8^, ^9^, ^10^, ^11^ ^]^ A large amount of attention has been directed especially to modifying the Cas proteins.^[^ ^12^, ^13^, ^14^, ^15^, ^16^, ^17^ ^]^ There is a considerable need for simpler approaches with no need for protein engineering. We therefore intend to target gRNAs for developing general and facile methods to manipulate CRISPR systems.

For the purpose of manipulating RNAs, chemical approaches based on postsynthetic chemistry are of great interest because they are easier to process.^[^ ^18^, ^19^, ^20^, ^21^, ^22^ ^]^ In previous studies, aldehydes have shown promise as modification reagents for nucleobases.^[^ ^23^, ^24^, ^25^, ^26^ ^]^ In particular, formaldehydes and glyoxals have been used to prevent the renaturation of thermally denatured DNA and also to study the secondary structure of RNA.^[^ ^27^, ^28^ ^]^ However, such linkages formed are labile and are readily broken even upon dialysis. We are therefore interested in behavior of compounds having multicarbonyl groups. From viewpoint of chemistry, the carbonyl group in such compounds is much more electrophilic than that in a simple aldehyde or ketone.^[^ ^29^ ^]^ In terms of structure, ninhydrin (1,2,3‐indantrione monohydrate) represents an ideal compound having three (potentially efficient in condensation reaction) carbonyl groups in one molecule.^[^ ^30^ ^]^ Though ninhydrin is traditionally used to detect amino acids and amines, its reaction with heterocyclic nucleobases has been reported.^[^ ^31^, ^32^ ^]^ The site of reaction is believed to be at the amino groups of those nucleobases, which act as 1,3‐binucleophiles. In this respect, the ninhydrin seems to have considerable potential, as yet unexploited, for modifying gRNA molecules.

**Figure 1:**
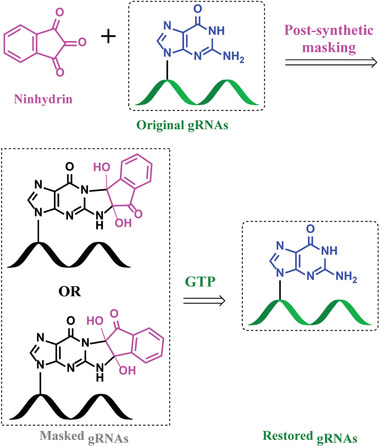
Schematic illustration of the design and workflow. Ninhydrin chemistry has been developed to manipulate gRNAs in CRISPR systems.

Different from previous researches, we here explore ninhydrin chemistry as a new strategy for modifying gRNA molecules. On the basis of our results, the reaction of ninhydrin with gRNAs proceeds readily in aqueous solution, with multiple tags attached to each strand (**Figure** **1** and Figure S1, Supporting Information). We further demonstrate that the ninhydrin‐gRNA adducts can be readily reverted to original gRNAs under mild conditions. We further show that ninhydrin chemistry is efficient in manipulating CRISPR systems. The CRISPR functions can be completely blocked by ninhydrin masking of gRNA. At a subsequent time, the CRISPR functions can be readily recovered without evident activity loss. Thus, ninhydrin chemistry is of value for the purpose of controlling structure and biological function of nucleic acids, and as aids to studying nucleic acid biology.

## 2 Results

### 2.1 Ninhydrin Chemistry to Control RNA‐Protein Interactions

**Figure 2:**
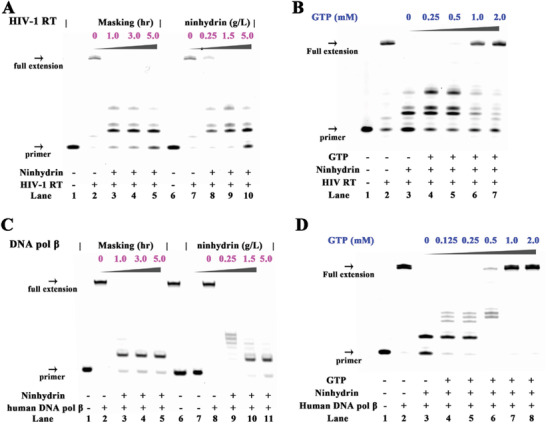
Ninhydrin chemistry to control RNA‐protein interactions. Reactions have been performed as described in the Experimental Section. All samples were tested in three biological replicates. Image of representative data is shown here. A) The influence of ninhydrin masking on RNA reverse transcription with HIV‐1 RT. Lanes 1, 6: no enzyme control; lanes 2–5: the RNA template has been masked with 5 g L^−1^ ninhydrin for different periods; lanes 7–10: the RNA template has been masked with different concentrations of ninhydrin for 5 h. B) The influence of GTP unmasking on RNA reverse transcription. C) The influence of ninhydrin masking on DNA replication with human DNA pol *β*. Lanes 1, 7: no enzyme control; lanes 2–5: the DNA template has been masked with 5 g L^−1^ ninhydrin for different periods; lane 6: DNA markers; lanes 8–11: the DNA template has been masked with different concentrations of ninhydrin for 5 h. D) The influence of GTP unmasking on DNA replication.

In many cases, the RNA‐protein interaction underlies the molecular basis for enzymatic recognition of substrate.^[^ ^33^, ^34^ ^]^ The reverse transcriptase (RT) is known to catalyze the formation of DNA products from RNA substrates.^[^ ^35^ ^]^ The masking of RNA substrate is expected to block the RT elongation. In this study, human immunodeficiency virus type 1 RT (HIV‐1 RT) and Moloney Murine Leukemia Virus RT (M‐MuLV RT) have been examined. The elongation scaffold was set up by assembling each RT with primer/template duplex (Dprimer1 and Rtemplate1 for scaffold 1 in Table S1, Supporting Information). Prior to ninhydrin masking, the RNA template in the duplex can be efficiently used to guide DNA synthesis (lanes 2 and 7 in **Figure** **2**A and Figure S2A, Supporting Information). The reverse transcription has been disabled by ninhydrin masking with both concentration‐ and time‐dependent manners (lanes 3–5 and 8–10 in Figure 2A and Figure S2A, Supporting Information).

For the ninhydrin‐modification reaction, the forward and reverse reactions are in balance at equilibrium (Figures S3 and S4, Supporting Information). Hence, a feasible strategy is to compete the ninhydrin group from RNA templates with a large excess of molecules with the similar structure to the guanine nucleobases. We therefore consider using guanosine‐5′‐triphosphate (GTP) because of its remarkable solubility in aqueous solutions. In this study, the ninhydrin‐masked RNA template has been treated with various amounts of GTP with heating to increase the reaction rates. Importantly, this blockage can be reversed with GTP unmasking (Figure 2B and Figure S2B, Supporting Information).

We further assessed the feasibility of using ninhydrin chemistry to manipulate DNA molecules. DNA replication is the process of copying DNA.^[^ ^36^ ^]^ The DNA polymerases (DNA pol) are known to catalyze this process. In this study, human DNA pol *β* and the *Bacillus stearothermophilus* DNA polymerase (*Bst* DNA pol) have been examined. The 5′‐Carboxyfluorescein (FAM)‐ labeled DNA primer is designed to target the 3′ end of DNA template (Dprimer1 and Dtemplate1 in Table S1, Supporting Information). The elongation scaffold was set up by assembling each DNA pol with the primer/template duplex (scaffold 2 in Table S1, Supporting Information). Prior to ninhydrin masking, the DNA template in the scaffold can be efficiently used to guide DNA synthesis. However after the template strands were masked with ninhydrin, the progress of each DNA pol was significantly blocked, leading to reduced DNA replication (Figure 2C and Figure S5A, Supporting Information). Moreover, the masking‐dependent inhibition has been reversed upon the GTP treatment (Figure 2D and Figure S5B, Supporting Information).

### 2.2 Ninhydrin Chemistry to Controlling CRISPR/Cas9 System

**Figure 3:**
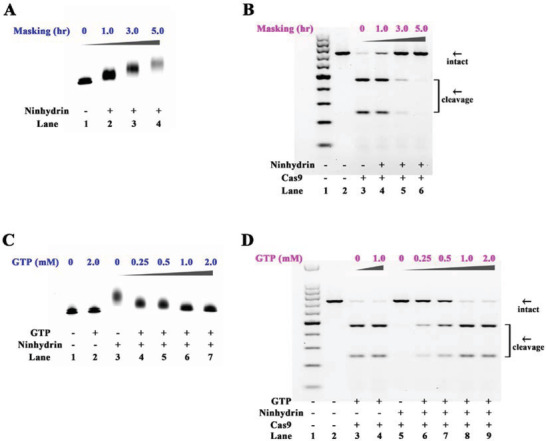
Ninhydrin chemistry to controlling CRISPR/Cas9 system. Reactions have been performed as described in the Experimental Section. All samples have been tested in three biological replicates. Image of representative data is shown here. Uncleaved t‐GFP1 DNA (702 bp) cut to shorter cleavage fragments (469 and 233 bp) are demonstrated. A) The ninhydrin masking of gRNA (gGFP1). The gGFP1 has been incubated with 5 g L^−1^ ninhydrin for different periods. B) The influence of ninhydrin masking on DNA cleavage. Lane 1: DNA markers; lane 2: no Cas9 control; lane 3: original gGFP1; lanes 4–6: gGFP1 with different masking levels. C) GTP unmasking of ninhydrin‐masked gGFP1. D) The influence of GTP unmasking on DNA cleavage. For (C) and (D), the masked gGFP1 (5 g L^−1^ ninhydrin, 5 h) has been incubated with different concentrations of GTP for 5 min.

The current study intends to use ninhydrin chemistry to control CRISPR systems, which are prevalent prokaryotic defense systems against foreign genetic elements.^[^ ^1^, ^3^ ^]^ The well‐adopted CRISPR/Cas9 system requires a gRNA moiety to recruit and direct the nuclease complexes.^[^ ^5^, ^6^ ^]^ The Cas9 protein then finds and cuts DNA targets at a specific site.^[^ ^37^ ^]^ Initially, in vitro transcription has been performed to synthesize different gRNAs targeting Green Fluorescent Protein (GFP) (gGFP1 in Table S1 and Figure S6, Supporting Information).^[^ ^38^ ^]^ We demonstrate that the ninhydrin masking significantly slows down the migration of the gRNA on a polyacrylamide gel (**Figure** **3**A). We next proceeded to test whether the ninhydrin masking can inhibit the function of CRISPR/Cas9. As demonstrated in Figure 3B and Figure S7A in the Supporting Information, the ninhydrin masking of gRNA (5 g L^−1^ ninhydrin, 3 h) evidently inhibits the DNA cleavage, and an almost complete inhibition has been observed with a further masking (5 g L^−1^ ninhydrin, 5 h).

We proceeded to test whether the ninhydrin groups can be removed to restore the CRISPR/Cas9 system. In this study, the ninhydrin‐masked gRNA has been treated with various amounts of GTP. The denaturing electrophoresis result demonstrated the successful unmasking of gRNA (Figure 3C). Moreover, the GTP unmasking has recovered the sequence‐specific DNA cleavage in a dose‐dependent manner (Figure 3D and Figure S7B, Supporting Information). We further demonstrate versatility of reversible ninhydrin chemistry for controlling CRISPR/Cas9 system using another gRNA (gGFP2 in Table S1 and Figures S8–S11, Supporting Information). These findings demonstrate that reversible ninhydrin chemistry is successful in achieving our objectives.

### 2.3 Ninhydrin Chemistry to Controlling CRISPR/Cas13a System

**Figure 4:**
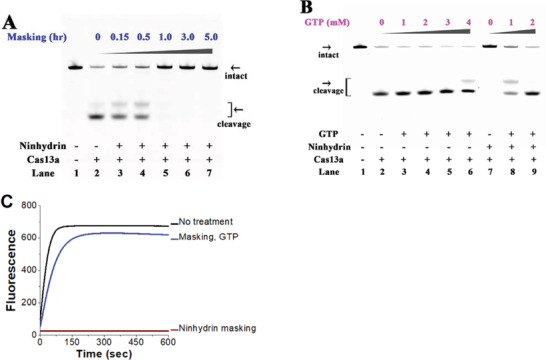
Ninhydrin chemistry to controlling CRISPR/Cas13a system. Reactions have been performed as described in the Experimental Section. All samples have been tested in three biological replicates. Image of representative data is shown here. A) The influence of ninhydrin masking on RNA cleavage. Lane 1: no Cas13a control; lane 2: original crRNA; lanes 3–7: crRNA with different masking levels. B) The influence of GTP unmasking on RNA cleavage. The ninhydrin‐masked crRNA (5 g L^−1^ ninhydrin, 5 h) has been treated with various concentrations of GTP for 5 min. C) Reversible control of the Cas13a collateral activity. The fluorescence of the sample versus time is shown.

We further examined our strategy in controlling RNA cleavage. The RNA‐activated RNA cleavage behavior of Cas13a (formerly C2c2) provides a powerful platform for numerous RNA targeting applications.^[^ ^39^, ^40^, ^41^, ^42^ ^]^ The Cas13a from *Leptotrichia buccalis* (Lbu Cas13a) is selected for our study.^[^ ^39^ ^]^ We first examined the performance of ninhydrin masking on the performance of CRISPR/Cas13a system. We observed significant masking‐dependent inhibition of the Cas13a cleavage (**Figure** **4**A). Importantly, the cleavage of target RNA was almost completely stopped by extensive ninhydrin masking (5 g L^−1^ ninhydrin for more than 1 h, lanes 5–7 in Figure 4A).

We expected the GTP unmasking to activate the CRISPR/Cas13a system. The results demonstrate that the GTP unmasking has recovered the sequence‐specific RNA cleavage in a concentration‐dependent manner (Figure 4B). Particularly, a substantial proportion of the target RNA was cleaved with the 1 × 10^−3^ m GTP treatment and that a 2 × 10^−3^ m GTP treatment almost completely recovered the Cas13a cleavage (lanes 8–9 in Figure 4B). Thus, the CRISPR/Cas13a system is efficiently controlled with the ninhydrin chemistry.

In bacteria, once Cas13a has recognized and cleaved its target RNA sequence as specified by the crRNA, it adopts an enzymatically “active” state rather than reverting to an “inactive” state.^[^ ^39^, ^41^ ^]^ As a result, the Cas13a will bind and cleave additional RNAs regardless of homology to crRNA. Our results consistently show that the Cas13a collateral activities were negligible in combination with the ninhydrin‐masked crRNA (wine line in Figure 4C), whereas the collateral activity of unmasked system was comparable with that of original system (blue and black lines in Figure 4C). All these results consistently indicate the good reversibility of ninhydrin chemistry for conditional control of CRISPR/Cas13a system.

### 2.4 The Study of Mechanisms of Action of Ninhydrin Chemistry

**Figure 5:**
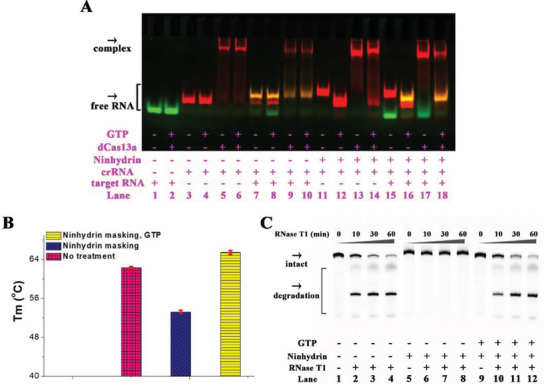
The study of mechanisms of action of ninhydrin chemistry. Reactions have been performed as described in the Experimental Section. All samples have been tested in three biological replicates. Image of representative data is shown here. A) The ninhydrin masking has little to no effect on the binding of the crRNA to dCas13a. The dCas13a has been incubated with cy3‐labeled crRNA probe with different treatments and/or FAM‐labeled target and the RNA‐protein complexes have been analyzed by 6% native PAGE. B) Representative melting profiles of the crRNA/target RNA with different treatments. The error bars reflect the standard deviation. C) Control of RNA cleavage with RNase T1. Lanes 1–4: original crRNA; lanes 5–8: the ninhydrin‐masked crRNA (5 g L^−1^ ninhydrin, 5 h); lanes 9–12: the ninhydrin‐masked crRNA after unmasking (8 × 10^−3^ m GTP).

We next explored potential mechanisms by which the ninhydrin chemistry functions to control the CRISPR systems. The CRISPR/Cas13a system was examined for proof of concept study. The ninhydrin masking might contribute to poor performance of CRISPR system through different mechanisms. To clarify this, we performed an electrophoretic mobility shift assay (EMSA) using the deactivated Cas13a (dCas13a).^[^ ^39^ ^]^ In this study, both the crRNA and complementary target RNA were labeled with different fluorophores (crRNA1‐cy3 and target1 in Table S1, Supporting Information). The results demonstrate that both the ss crRNA and the crRNA/target duplex can bind the dCas13a protein (lanes 5 and 9 in **Figure** **5**A). The EMSA results also indicate that the ninhydrin modification significantly affected the hybridization between the crRNA and the complementary target (lanes 7 and 15 in Figure 5A). Moreover, the GTP unmasking efficiently reversed this trend (lanes 15 and 16 in Figure 5A). Surprisingly, the ninhydrin modification has little to no effect on the binding of the crRNA to dCas13a (lanes 13 and 17 in Figure 5A). On the basis of these results, the ninhydrin‐masked crRNA can still associate with the Cas13a but fails to form a cleavage‐competent complex.

The UV melting assay further indicated that the ninhydrin masking significantly blocked the interactions between the crRNA and the target RNA (Figure 5B and Figure S12, Supporting Information). Specifically, the ninhydrin‐masked sample showed an evidently lower *T*_m_ as compared with that of the unreacted control (red and black lines in Figure S12, Supporting Information). Moreover, the GTP treatment is very efficient for recovering the RNA hybrids (blue line in Figure S12, Supporting Information).

We proceeded to test whether ninhydrin chemistry can be used to protect gRNA from degradation. The RNase T1 has been evaluated in this study. The results demonstrate that the RNase T1 efficiently cleaves ss RNA substrates (lanes 1–4 in Figure 5C). When ss RNA substrate was masked with ninhydrin (lanes 5–8 in Figure 5C), only small fractions of RNA digestion was observed. When the GTP unmasking was performed, the RNA cleavage was performed at a rate comparable with that observed for the original ss RNA (lanes 9–12 in Figure 5C).

## 3 Discussion

An amount of attention has been directed to the use of formaldehyde for modifying nucleobases.^[^ ^23^, ^25^ ^]^ Our designed strategy is based on the knowledge that ninhydrin has a 1,2,3‐tricarbonyl group that exhibits high electrophilicity. In this molecule, the carbon atom of a carbonyl bears a partial positive charge enhanced by neighboring electron withdrawing carbonyl groups. It can react potentially with a variety of nucleophiles, including those nucleobases with exocyclic amino groups.^[^ ^31^, ^32^ ^]^ The covalent adducts of gRNAs with multicyclic ninhydrin would further block their access to other biomolecules, such as Cas proteins. Moreover, ninhydrin chemistry meets another important requirement: it may be released during a competitive deprotection step. All of these features make ninhydrin chemistry attractive as a new chemical strategy to controlling CRISPR systems.

In the current study, both CRISPR/Cas9 and CRISPR/Cas13a systems were rendered inactive by installation of ninhydrin groups on gRNAs to prevent their hybridization to the complementary target sequences.^[^ ^33^ ^]^ Brief GTP treatment removes the ninhydrin groups, enables efficient sequence‐specific nucleic acid cleavage. In addition to controlling CRISPR functions, the ninhydrin chemistry maintains other desired characteristics such as increased nuclease resistance. Future studies and development of the ninhydrin chemistry must include testing it within living systems.

## 4 Conclusions

In summary, we demonstrate the effectiveness of ninhydrin chemistry for conditional control of RNA‐guided nucleic acid cleavage. Our strategy will therefore be potentially beneficial in future chemical and biological applications.

## 5 Experimental Section

##### Materials

All oligonucleotide sequences are provided in Table S1 in the Supporting Information. The ninhydrin (product number: 151173) and all other chemicals were purchased from Sigma‐Aldrich (Shanghai, China). The oligonucleotides were synthesized from TaKaRa company (Dalian, China). The HIV‐1 RT, Recombinant, *E. coli* (product number: 382129) was purchased from Millipore Corporation (Merck KGaA, Germany). Human DNA pol *β* (product number: 1077) was purchased from CHIMERx (Madison, WI, USA). The M‐MuLV RT (product number: M0253) and *Bst* DNA pol, Large Fragment (product number: M0275), the Cas9 Nuclease, Streptococcus pyogenes (product number: M0646) were purchased from New England Biolabs, Inc. (USA). The nucleic acid stains Super GelRed (NO.: S‐2001) was purchased from US Everbright Inc. (Suzhou, China). The GTP (product number: 4042) was purchased from TaKaRa Shuzo Co. Ltd. (Tokyo, Japan). Transcript Aid T7 High Yield Transcription kit (product number: K0441) and Glycogen (product number: R0561) were purchased from Thermo Fisher Scientific. The DNA Clean and Concentrator‐5 kit (product number: D4014) was purchased from Zymo Research Corp. The pH was measured with Mettler Toledo, FE20‐Five Easy pH (Mettler Toledo, Switzerland). The concentration of nucleic acids was quantified by NanoDrop 2000c (Thermo Scientific, USA). The Circular Dichroism (CD) experiments were performed on a Jasco‐810 spectropolarimeter (Jasco, Easton, MD, USA) equipped with a Peltier temperature controller.

##### The Masking of RNA with Ninhydrin

The ninhydrin stock solution was prepared in deionized water at 20 g L^−1^. The 20 µL reaction mixture containing 10 µg RNA (10 µL), the ninhydrin stock solution (x µL), and nuclease‐free water (10 − x µL) was incubated at 37 °C with shaking. The ninhydrin‐masked RNA was purified with ethanol precipitation.

##### Reversion of Ninhydrin‐Masked RNA

The ninhydrin‐masked RNA (10 µg) was incubated with various concentrations of GTP at 70 °C for 5 min and was cooled quickly to 4 °C. This obtained sample was used without further purification.

##### Denaturing PAGE Analysis

The denaturing polyacrylamide gel electrophoresis (PAGE) analysis was run in a temperature‐controlled vertical electrophoretic apparatus (DYCZ‐22A, Liuyi Instrument Factory, Beijing, China). The acrylamide concentration of the separating gel was 20 % (19:1 monomer to bis ratio). About 100 ng of nucleic acids were loaded on the gel. Electrophoresis was run at 10 °C for 4.0 h at 400 V. After electrophoresis, the oligomers in the gel were visualized using a Pharos FX Molecular imager (Bio‐Rad, USA) in the fluorescence mode (*λ*_ex_ = 488 or 590 nm).

##### Ninhydrin Chemistry to Controlling Reverse Transcription

For HIV‐1 RT, this reaction was performed in 1 × reaction buffer, which contained 50 × 10^−3^ m Tris‐HCl (pH 8.3), 6 × 10^−3^ m MgCl_2_, and 40 × 10^−3^ m KCl.

For ninhydrin blockage assay, the 5′‐FAM‐labeled DNA primer (Dprimer1) was mixed with RNA templates (Rtemplate1) with different masking levels. The extension scaffold (scaffold 1 in Table S1, Supporting Information) was then preincubated with excess HIV‐1 RT in 1 × reaction buffer to make the enzyme: scaffold complex. The reaction was started by rapid mixing of equal volumes of the enzyme: scaffold complex with a solution containing twofold concentrations of dNTPs in 1 × reaction buffer. The final concentration of each component for a typical 10 µL reaction is as follow: 2.0 U HIV‐1 RT, 100 × 10^−9^ m Dprimer1, 500 × 10^−9^ m Rtemplate1, and 200 × 10^−6^ m dNTPs. Incubation was performed at 37 °C for 2 h. The following quenching and electrophoresis steps are similar to the progression steps of the procedure described above (DNA replication).

For GTP activation assay, the extension scaffold was prepared in reaction buffer containing the Dprimer1 and Rtemplate1 with the desired masking level (5 g L^−1^ ninhydrin, 5 h), followed by the addition of different concentrations of GTP. The preparation was heated at 70 °C for 5 min and then cooled to room temperature for 5 min. The following procedure is similar to the above one.

##### Ninhydrin Chemistry to Controlling DNA Replication

For human DNA pol *β*, this assay was performed in 1 × reaction buffer, which contained 50 × 10^−3^ m Tris‐HCl (pH 8.7), 100 × 10^−3^ m KCl, 10 × 10^−3^ m MgCl_2_, 0.4 mg mL^−1^ bovine serum albumin (BSA), 1.0 × 10^−3^ m dithiothreitol, and 15% v/v glycerol.

For ninhydrin blockage assay, the 5′‐FAM‐labeled DNA primer (Dprimer1) was annealed with DNA templates (Dtemplate1) with different masking levels. The prepared extension scaffold (scaffold 2 in Table S1, Supporting Information) was then preincubated with human DNA pol *β* in 1 × reaction buffer to make the enzyme: scaffold complex. The reaction was started by rapid mixing of equal volumes of the enzyme: scaffold complex with an solution containing twofold concentrations of dNTPs in 1 × reaction buffer. The final concentration of each component for a typical 10 µL reaction is as follow: 1.0 U human DNA pol *β*, 100 × 10^−9^ m Dprimer1, 200 × 10^−9^ m Dtemplate1, and 200 × 10^−6^ m dNTPs. Incubation was performed at 37 °C for 2 h. The reactions were quenched by adding 4 × v:v of stop buffer (95% formamide, 25 × 10^−3^ m Ethylenediaminetetraacetic acid (EDTA) at pH 8.0), followed by immediate heating at 90 °C for 5 min. After cooling down to 4 °C, products were analyzed by denaturing electrophoresis (20% polyacrylamide, 19:1). The starting Dtemplate1 without ninhydrin treatment was used as the control.

For GTP activation assay, the extension scaffold was prepared in reaction buffer containing the Dprimer1 and Dtemplate1 with the desired masking level (5 g L^−1^ ninhydrin, 5 h), followed by the addition of different concentrations of GTP. The preparation was heated at 70 °C for 5 min and then cooled to room temperature for 5 min. The following procedure is similar to the above one.

##### In Vitro Transcription and Purification of gRNAs

In vitro transcription with the T7 RNA polymerase is performed to synthesize various gRNAs.^[^ ^38^ ^]^ Transcription reactions were performed at 37 °C for 4.0 h in 1 × Transcript aid reaction buffer according to the manufacturer protocol. Following DNA degradation using DNase I at the end of the transcription, the transcribed RNA products were then purified using the NaOAc/phenol/chloroform method. The purified gRNA was resuspended in RNase‐free H_2_O.

##### Ninhydrin Chemistry to Controlling CRISPR/Cas9 System

In vitro Cas9 cleavage assay was performed in 1 × NEBuffer 3.1, which contained 100 × 10^−3^ m NaCl, 50 × 10^−3^ m Tris‐HCl, 10 × 10^−3^ m MgCl_2_, and 100 µg mL^−1^ BSA at pH 7.9@25 °C. Briefly, 60 ng of gRNA, 100 ng of target DNA substrate, 1.0 µg of BSA, and Cas9 (1.0 U) were incubated with 10 µL of 1 × Cas9 buffer in RNase free water at 37 °C according to the manufacturer's protocol.

For ninhydrin blockage assay, the gRNA (60 ng) with different masking levels was mixed with DNA amplicon substrate (100 ng) and subjected to an enzymatic digest with Cas9 for 20 h. Reactions were quenched by adding Sodium dodecyl sulfate (SDS) containing loading dye and loaded onto a 1.5% agarose gel containing 1.5 × Super GelRed for visualization.

For GTP activation assay, the ninhydrin‐modified gRNA (5 g L^−1^, 5 h) was incubated with various concentrations of GTP at 70 °C for 5 min, and the mixture was cooled to room temperature for 5 min. The following procedure is similar to the above one.

##### Cas13a Expression and Purification

The wild type and mutated LbuCas13a proteins were expressed according to a previous report.^[^ ^39^ ^]^

##### Ninhydrin Chemistry to Controlling CRISPR/Cas13a System

In vitro Cas13a cleavage assay has been performed in 1 × Cas13a buffer in RNase free water, which contained 20 × 10^−3^ m 4‐(2‐hydroxyethyl)‐1‐piperazineethanesulfonic acid (HEPES), 50 × 10^−3^ m KCl, 5 × 10^−3^ m MgCl_2_, and 5% glycerol at pH 6.8@25 °C.

For ninhydrin blockage assay, the crRNA with different masking levels was mixed with RNA targets and subjected to the Cas13a cleavage. The final concentration of each component was as follows: 45 × 10^−9^ m purified Cas13a protein, 22.5 × 10^−9^ m crRNA, 150 × 10^−9^ m 5′‐end labeled target RNA, and 0.5 U RiboLock RNase inhibitor (Thermo Fisher Scientific). Reactions were allowed to proceed for 2 h at 37 °C (unless otherwise indicated). Reactions were analyzed on a denaturing 20% polyacrylamide gel (400 V, 30 min).

For GTP activation assay, the ninhydrin‐modified crRNA (5 g L^−1^, 5 h) was incubated with various concentrations of GTP at 70 °C for 5 min, and the mixture was cooled to room temperature for 5 min. The following procedure is similar to the above one.

##### Ninhydrin Chemistry to Controlling Cas13a Collateral‐Cleavage

This assay was performed with 45 × 10^−9^ m purified LbuCas13a, 22.5 × 10^−9^ m crRNA, 22.5 × 10^−9^ m target RNA, 125 × 10^−9^ m quenched fluorescent RNA reporter (reporter1 in Table S1, Supporting Information), and 0.5 U RiboLock RNase inhibitor, in 1 × Cas13a buffer.^[^ ^39^ ^]^ The detection is conducted at room temperature with a 1 cm path‐length cell. For kinetic measurement, RNA reporter at a final concentration of 125 × 10^−9^ m was added at time (*t*) = 0. Reactions were allowed to proceed for 1 h measured every 1 s. The excitation and emission wavelengths are set to 496 and 520 nm. Slit width: excitation = 10 nm; emission = 10 nm.

##### EMSA Assay

This assay was performed in 1 × EMSA buffer, which contained 20 × 10^−3^ m HEPES, 50 × 10^−3^ m KCl, 10 µg mL^−1^ BSA, 100 µg mL^−1^ yeast tRNA, 0.01% Igepal CA‐630, and 5% glycerol at pH 6.8@25 °C.^[^ ^39^ ^]^ The 5′‐cy3 labeled crRNA and/or 5′‐FAM labeled target RNA were incubated with the dCas13a for 10 min at 37 °C. Samples were then resolved by 6% native PAGE at 4 °C (0.5 ×  Tris/Borate/EDTA (TBE) buffer). Electrophoresis was run at 6 °C for 1 h at 300 V and 1 h at 200 V. After electrophoresis, in gel targets were analyzed using a Pharos FX Molecular imager (Bio‐Rad, USA) in the fluorescence mode (*λ*_ex_ = 488 and 590 nm).

##### Ninhydrin Chemistry to Controlling RNA Cleavage

This assay was performed in 1 × reaction buffer in Polymerase Chain Reaction (PCR) grade water, which contained 50 × 10^−3^ m Tris‐HCl and 2 × 10^−3^ m EDTA at pH 7.5@25 °C. The 5′‐cy3 labeled ss RNA (crRNA‐cy3, 100 ng) with the desired masking level (20 g L^−1^ ninhydrin, 5 h) was incubated in the presence of 1.5 U RNase T1 in a reaction volume of 10 µL at 37 °C for various periods. Reactions were analyzed on a denaturing 20% polyacrylamide gel (350 V, 1.0 h).

For GTP activation assay, the ninhydrin‐modified crRNA‐cy3 was incubated with 8 × 10^−3^ m GTP at 80 °C for 5 min and the mixture was cooled to room temperature for 5 min. The following procedure is similar to the above one.

## Conflict of Interest

The authors declare no conflict of interest.

## Author Contributions

T.T. conceived the original idea, designed the study and led the project. S.‐R.W. and H.‐Y.H. performed most of the assays. J.L., and P.Y. contributed to protein expression and purification. L.W., L.‐Y.W., and W.X. contributed to chemical synthesis. T.T.; X.Z.; and S.‐R.W. performed data analysis and wrote the manuscript. S.‐R.W. and H.‐Y.H. share co‐first authorship for this work. All the authors provided feedback on the study and on the manuscript.

## Supporting information

Supporting Information
