# Supplementary material for: The Manipulation of RNA‐Guided Nucleic Acid Cleavage with Ninhydrin Chemistry
Source: Adv Sci (Weinh). 2020 May 26;7(13):1903770. doi: 10.1002/advs.201903770 (PMC7341091; doi:10.1002/advs.201903770)
Supplement: Supplementary file 1 — Supporting Information [file ADVS-7-1903770-s001.pdf]

Supporting Information

The manipulation of RNA-guided nucleic acid cleavage with ninhydrin chemistry

Shao-Ru Wang[1,2,3,5], Hai-Yan Huang[1,5], Jian Liu[4], Lai Wei[1], Ling-Yu Wu[1], Wei Xiong[1], Ping Yin[4], Tian Tian[1,3,]*, Xiang Zhou[1,3]*

Email: ttian@whu.edu.cn, xzhou@whu.edu.cn

Table of contents

	Materials and general methods	Page S1
Table S1	Sequences of oligomers used in the current study	Page S2
Figure S1	Schematic illustration of the design and workflow	Page S3
Figure S2	Conditional control of RNA reverse transcription	Page S4
Figure S3	Schematic illustration of the design and workflow	Page S5
Figure S4	Schematic illustration of the design and workflow	Page S6
Figure S5	Conditional control of DNA replication	Page S7
Figure S6	Schematic illustration of target sites of the gRNA in the GFP gene	Page S8
Figure S7	Ninhydrin chemistry to controlling CRISPR/Cas9 system	Page S9
Figure S8	Schematic illustration of target sites of the gRNA in the GFP gene	Page S10
Figure S9	Ninhydrin chemistry to controlling CRISPR/Cas9 system	Page S11
Figure S10	Ninhydrin chemistry to controlling CRISPR/Cas9 system	Page S12
Figure S11	Ninhydrin chemistry to controlling CRISPR/Cas9 system	Page S13
Figure S12	UV melting studies	Page S14

Materials and general methods

Materials

All oligonucleotide sequences are provided in Table S1. The ninhydrin (product# 151173) and all other chemicals were purchased from Sigma-Aldrich (Shanghai, China). The oligonucleotides were synthesized from TaKaRa company (Dalian, China). The HIV-1 RT, Recombinant, *E. coli* (product number: 382129) was purchased from EMD Millipore Corporation (Merck KGaA, Germany). Human DNA pol β (product number. 1077) was purchased from CHIMERx (Madison, WI, USA). The M-MuLV RT (product number: M0253) and *Bst* DNA pol, Large Fragment (product number: M0275), the Cas9 Nuclease, Streptococcus pyogenes (product# M0646), the ribonucleotide solution mix (NTPs) and deoxy-ribonucleoside triphosphates (dNTPs) were purchased from New England Biolabs, Inc. (USA). The nucleic acid stains Super GelRed (NO.: S-2001) was purchased from US Everbright Inc. (Suzhou, China). The GTP (product# 4042) and the Pyrobest™ DNA Polymerase were purchased from TaKaRa Shuzo Co. Ltd. (Tokyo, Japan). Transcript Aid T7 High Yield Transcription kit (product# K0441) and Glycogen (product# R0561) were purchased from Thermo Fisher Scientific. The DNA Clean & Concentrator™-5 kit (product# D4014) was purchased from Zymo Research Corp. The pH was measured with Mettler Toledo, FE20-Five Easy™ pH (Mettler Toledo, Switzerland). The concentration of nucleic acids was quantified by NanoDrop 2000c (Thermo Scientific, USA). The CD experiments were performed on a Jasco-810 spectropolarimeter (Jasco, Easton, MD, USA) equipped with a Peltier temperature controller.

Table S1. Sequences of oligomers used in the current study

<table>
<tr><th>Oligomer</th><th>Sequence(from 5’to 3’)</th><th colspan="2">Construct</th></tr>
<tr><td>Rtemplate1</td><td>5’-AAGUCGAUCUCAGUGCAGUACAAGUAAUCCAU-3’</td><td rowspan="2">scaffold 1</td><td></td></tr>
<tr><td>Dprimer1</td><td>5’-FAM-ATGGATTACTT-3’</td><td rowspan="2">scaffold 2</td></tr>
<tr><td>Dtemplate1</td><td>5’-AAGCTGATCTCGATGACGTACAAGTAATCCAT-3’</td><td></td></tr>
<tr><td>gGFP-1F</td><td>5’-TCTAATACGACTCACTATAGGGATGCCGTTCTTCTGCTTGTGTTTTAGAGCTAGAAATAGCA-3’</td><td rowspan="2">For gRNA construct (gGFP1)</td><td></td></tr>
<tr><td>gRNA-R</td><td>5’-AAAAGCACCGACTCGGTGCCACTTTTTCAAGTTGATAACGGACTAGCCTTATTTTAACTTGCTATTTCTAGCTCTAAAAC-3’</td><td rowspan="2">For gRNA construct (gGFP2)</td></tr>
<tr><td>gGFP-2F</td><td>5’-TCTAATACGACTCACTATAGGGTGGTGCAGATGAACTTCAGTTTTAGAGCTAGAAATAGCA-3’</td><td></td></tr>
<tr><td>gGFP1</td><td>5’-GGGAUGCCGUUCUUCUGCUUGUGUUUUAGAGCUAGAAAUAGCAAGUUAAAAUAAGGCUAGUCCGUUAUCAACUUGAAAAAGUGGCACCGAGUCGGUGCUUUU-3’</td><td colspan="2">transcribed RNA</td></tr>
<tr><td>gGFP2</td><td>5’-GGGUGGUGCAGAUGAACUUCAGUUUUAGAGCUAGAAAUAGCAAGUUAAAAUAAGGCUAGUCCGUUAUCAACUUGAAAAAGUGGCACCGAGUCGGUGCUUUU-3’</td><td colspan="2">transcribed RNA</td></tr>
<tr><td>t-GFP-1F</td><td>5’-GAGGAGCTGTTCACCGGG-3’</td><td colspan="2" rowspan="2">For PCR of t-GFP1</td></tr>
<tr><td>t-GFP-1R</td><td>5’-CTTGTACAGCTCGTCCATGC-3’</td></tr>
<tr><td>t-GFP-2F</td><td>5’-GACGTAAACGGCCACAAGTTC-3’</td><td colspan="2" rowspan="2">For PCR of t-GFP2</td></tr>
<tr><td>t-GFP-2R</td><td>5’-GGGGTGTTCTGCTGGTAGTG-3’</td></tr>
<tr><td>crRNA1</td><td>5’-GAUUUAGACUACCCCAAAAACGAAGGGGACUAAAACUAGAUUGCUGUUCUACCAAGUAAUCCAU-3’</td><td colspan="2">RNA</td></tr>
<tr><td>target1</td><td>5’-FAM-UUACUUGGUAGAACAGCAAUCUA-3’</td><td colspan="2">RNA</td></tr>
<tr><td>reporter1</td><td>5’-FAM-UUUUU-BHQ1-3’</td><td colspan="2">RNA</td></tr>
<tr><td>crRNA1-cy3</td><td>5’-cy3-GAUUUAGACUACCCCAAAAACGAAGGGGACUAAAACUAGAUUGCUGUUCUACCAAGUAAUCCAU-3’</td><td colspan="2">RNA</td></tr>
</table>

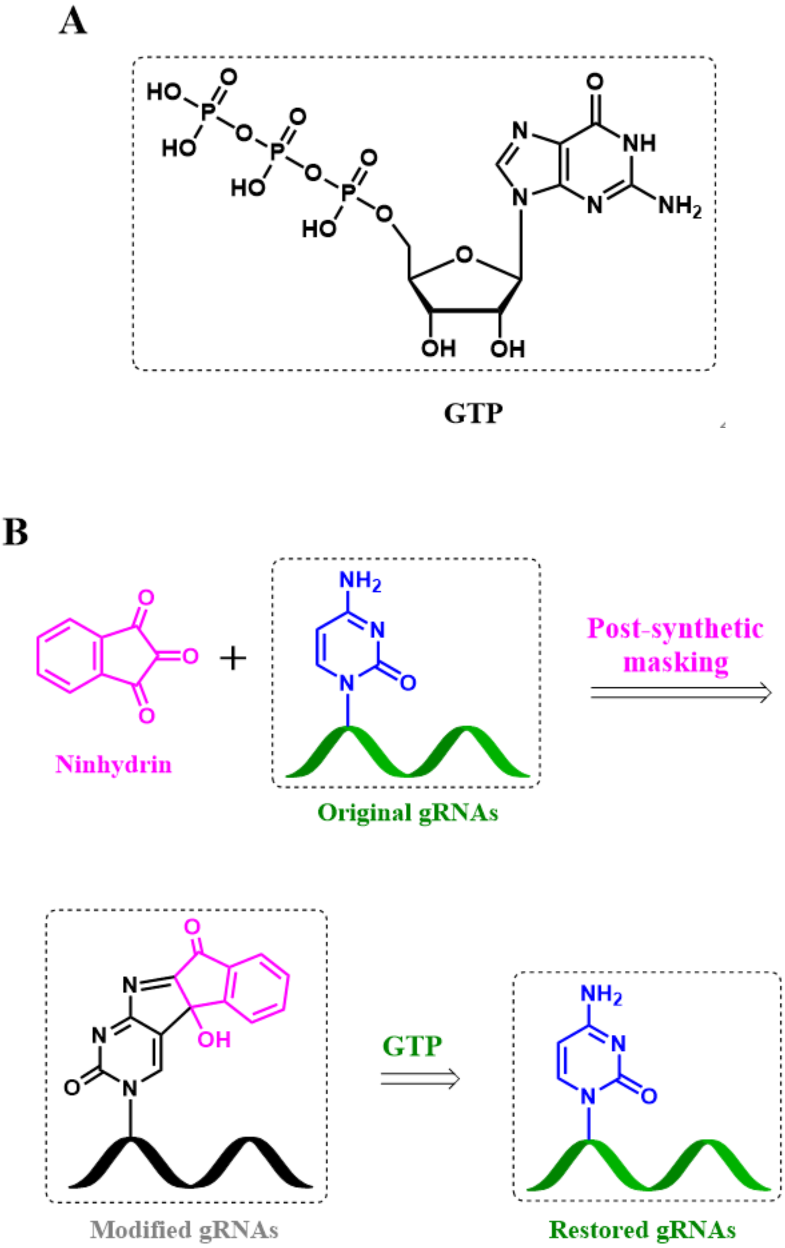

Figure S1: Schematic illustration of the design and workflow (**A**) Chemical structure of GTP. (**B**) Reversible masking of cytosine in gRNAs.

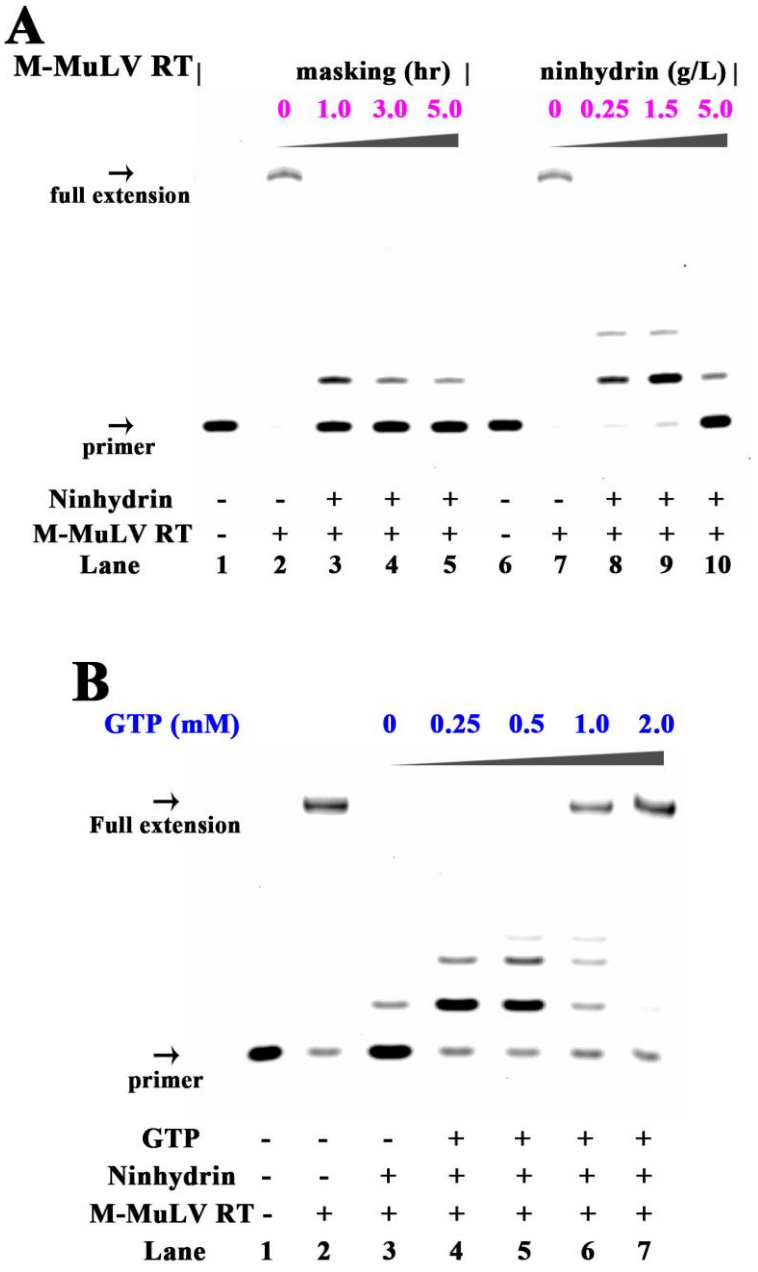

Figure S2. Conditional control of RNA reverse transcription Reactions have been performed as described in the Experimental Section. All samples were tested in three biological replicates. Image of representative data is shown here. (**A**) The influence of ninhydrin masking on RNA reverse transcription with the M-MuLV RT. The RNA template was masked with 5 g/L ninhydrin for different periods or masked with different concentrations of ninhydrin for 5 hr. (**B**) The influence of GTP unmasking on RNA reverse transcription with the M-MuLV RT.

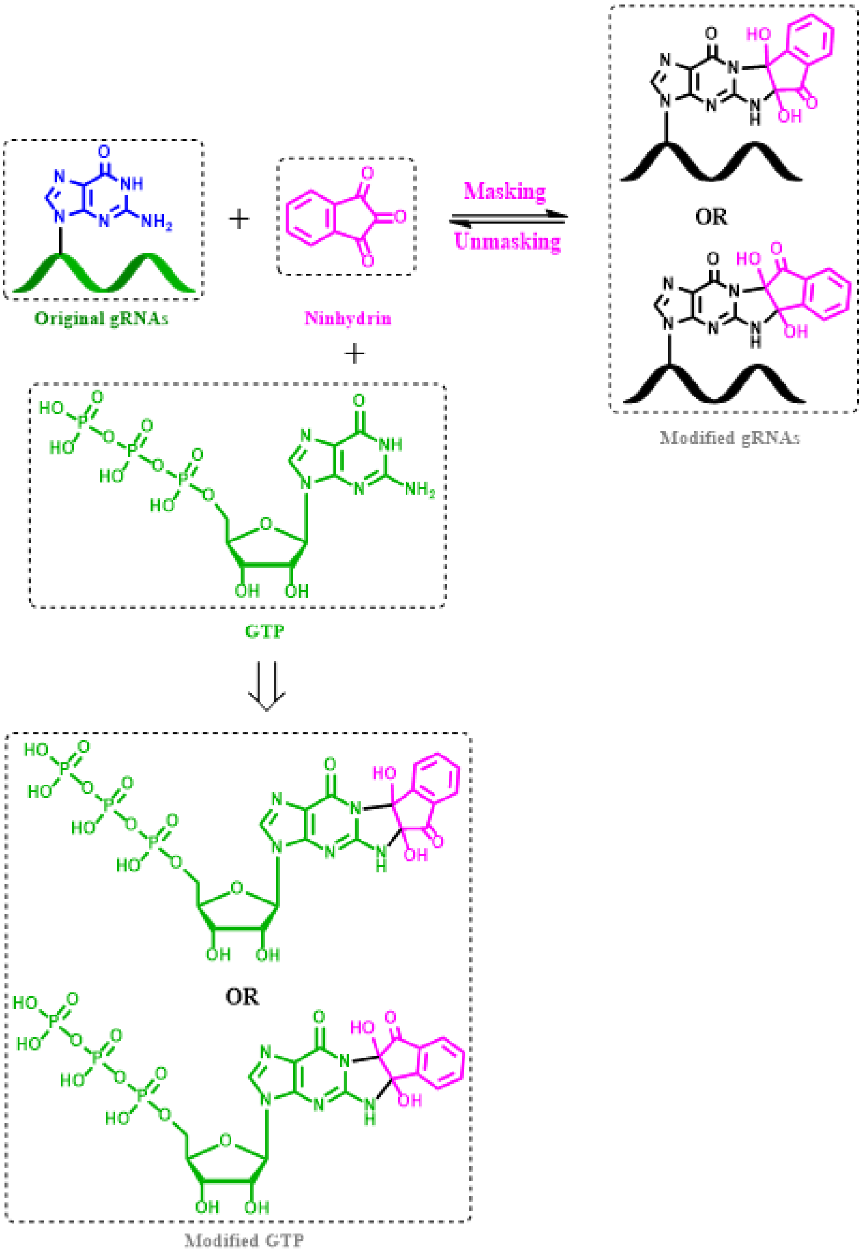

Figure S3: Schematic illustration of the design and workflow For the ninhydrin masking reactions, the forward and reverse reactions are in balance at equilibrium. For ninhydrin chemistry, a large excess of GTP has been used to compete the ninhydrin group from nucleic acid strands.

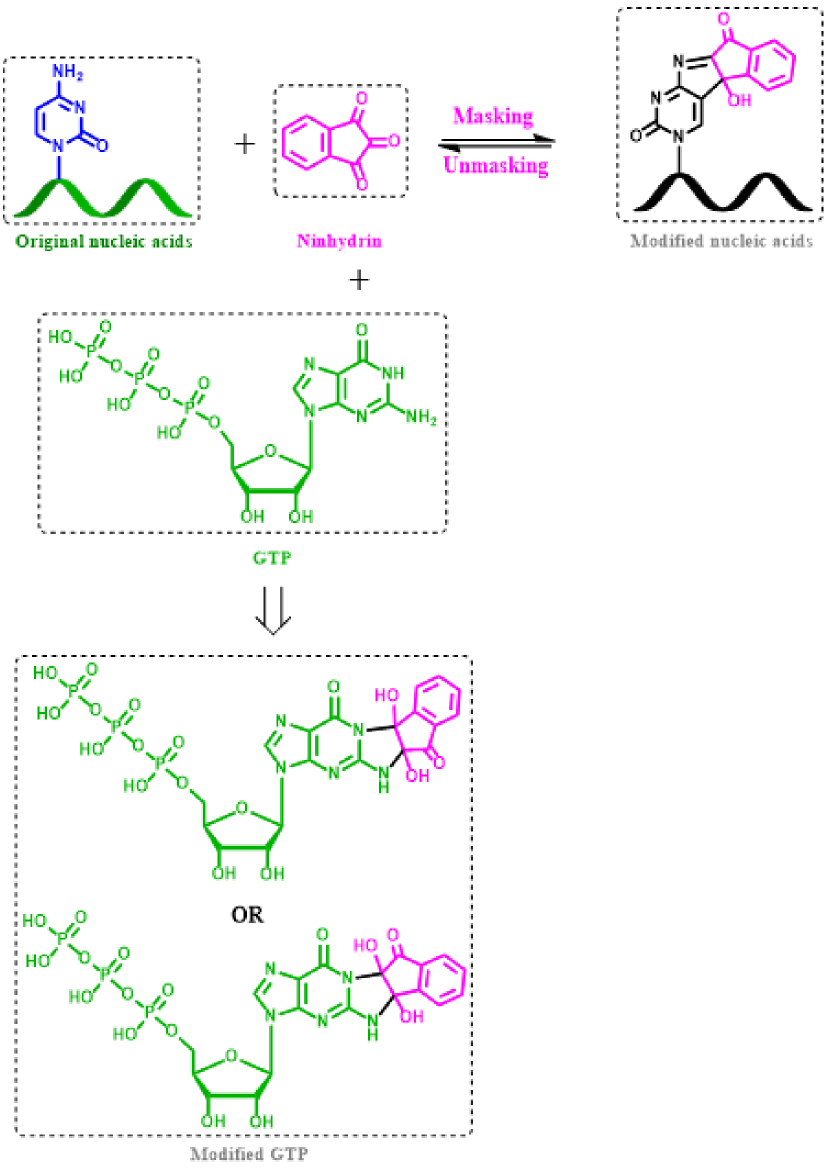

Figure S4: Schematic illustration of the design and workflow For the ninhydrin masking reactions, the forward and reverse reactions are in balance at equilibrium. For ninhydrin chemistry, a large excess of GTP has been used to compete the ninhydrin group from nucleic acid strands.

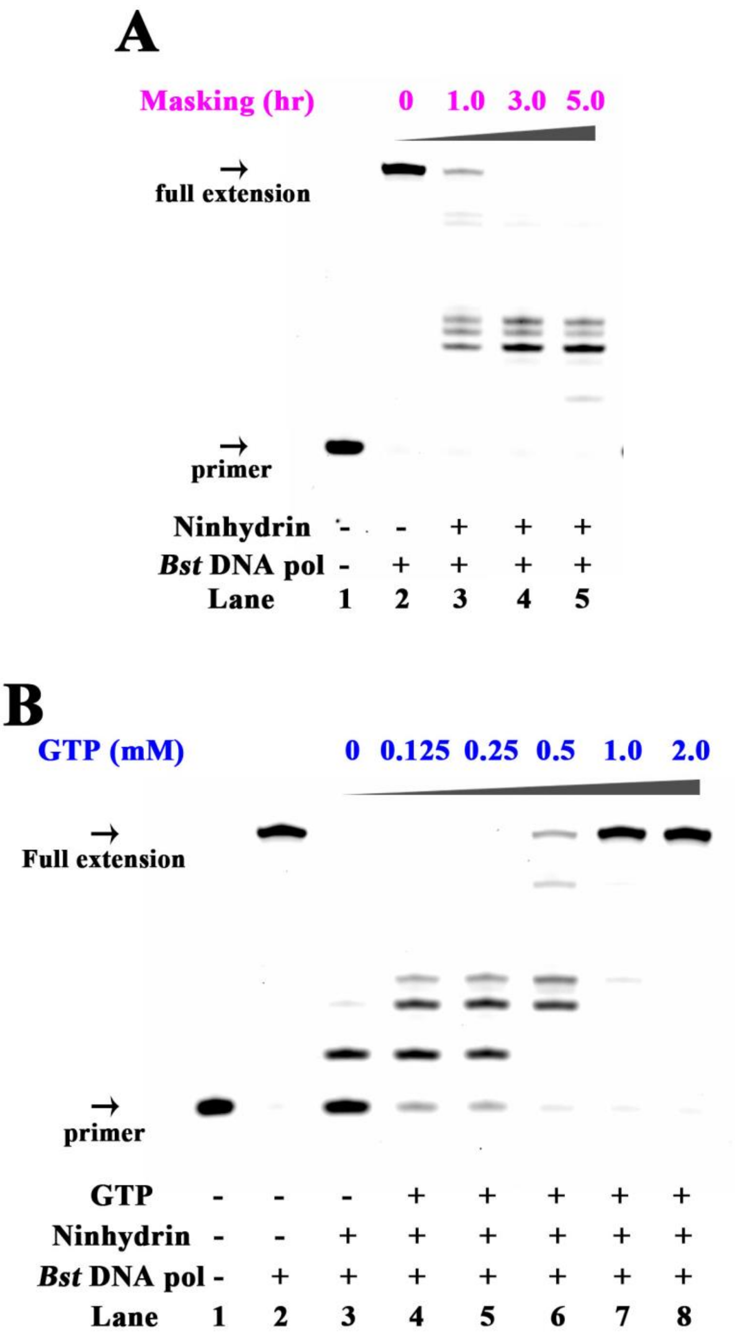

Figure S5. Conditional control of DNA replication Reactions have been performed as described in the Experimental Section. All samples were tested in three biological replicates. Image of representative data is shown here. (**A**) The influence of ninhydrin masking on DNA replication with *Bst* DNA pol. The DNA template has been masked with 5 g/L ninhydrin for different periods. (**B**) The influence of GTP unmasking on DNA replication with *Bst* DNA pol.

A

GAGGAGCTGTTCACCGGGGTGGTGCCCATCCTGGTCGAGCTGGACGGCGACGTAAA
CGGCCACAAGTTCAGCGTGTCCGGCGAGGGCGAGGGCGATGCCACCTACGGCAAGCT
GACCCTGAAGTTCATCTGCACCACCGGCAAGCTGCCCGTGCCCTGGCCCACCCTCGTG
ACCACCCTGACCTACGGCGTGCAGTGCTTCAGCCGCTACCCCGACCACATGAAGCAG
CACGACTTCTTCAAGTCCGCCATGCCCGAAGGCTACGTCCAGGAGCGCACCATCTTCT
TCAAGGACGACGGCAACTACAAGACCCGCGCCGAGGTGAAGTTCGAGGGCGACACC
CTGGTGAACCGCATCGAGCTGAAGGGCATCGACTTCAAGGAGGACGGCAACATCCTG
GGGCACAAGCTGGAGTACAACTACAACAGCCACAACGTCTATATCATGGCCGACAAG
CAGAAGAACGGCA^TCAAGGTGAACTTCAAGATCCGCCACAACATCGAGGACGGCA
GCGTGCAGCTCGCCGACCACTACCAGCAGAACACCCCCATCGGCGACGGCCCCGTGC
TGCTGCCCGACAACCACTACCTGAGCACCCAGTCCGCCCTGAGCAAAGACCCCAACG
AGAAGCGCGATCACATGGTCCTGCTGGAGTTCGTGACCGCCGCCGGGATCACTCTCG
GCATGGACGAGCTGTACAAG

B

GACGTAAACGGCCACAAGTTCAGCGTGTCCGGCGAGGGCGAGGGCGATGCCACCTA
CGGCAAGCTGACCCTGAAGTTCATCTGCACCACCGGCAAGCTGCCCGTGCCCTGGCCC
ACCCTCGTGACCACCCTGACCTACGGCGTGCAGTGCTTCAGCCGCTACCCCGACCACA
TGAAGCAGCACGACTTCTTCAAGTCCGCCATGCCCGAAGGCTACGTCCAGGAGCGCA
CCATCTTCTTCAAGGACGACGGCAACTACAAGACCCGCGCCGAGGTGAAGTTCGAGG
GCGACACCCTGGTGAACCGCATCGAGCTGAAGGGCATCGACTTCAAGGAGGACGGCA
ACATCCTGGGGCACAAGCTGGAGTACAACTACAACAGCCACAACGTCTATATCATGG
CCGACAAGCAGAAGAACGGCA^TCAAGGTGAACTTCAAGATCCGCCACAACATCGA
GGACGGCAGCGTGCAGCTCGCCGAC**CACTACCAGCAGAACACCCC**

Figure S6. Schematic illustration of target sites of the gRNA in the GFP gene The purple underlines indicate the target loci of gRNA (gGFP1) and black caret shows the cleavage sites. Two GFP targets (t-GFP1 and t-GFP2) were amplified from pEGFP-C1 vector. (**A**) The demonstration of t-GFP1. (**B**) The demonstration of t-GFP2.

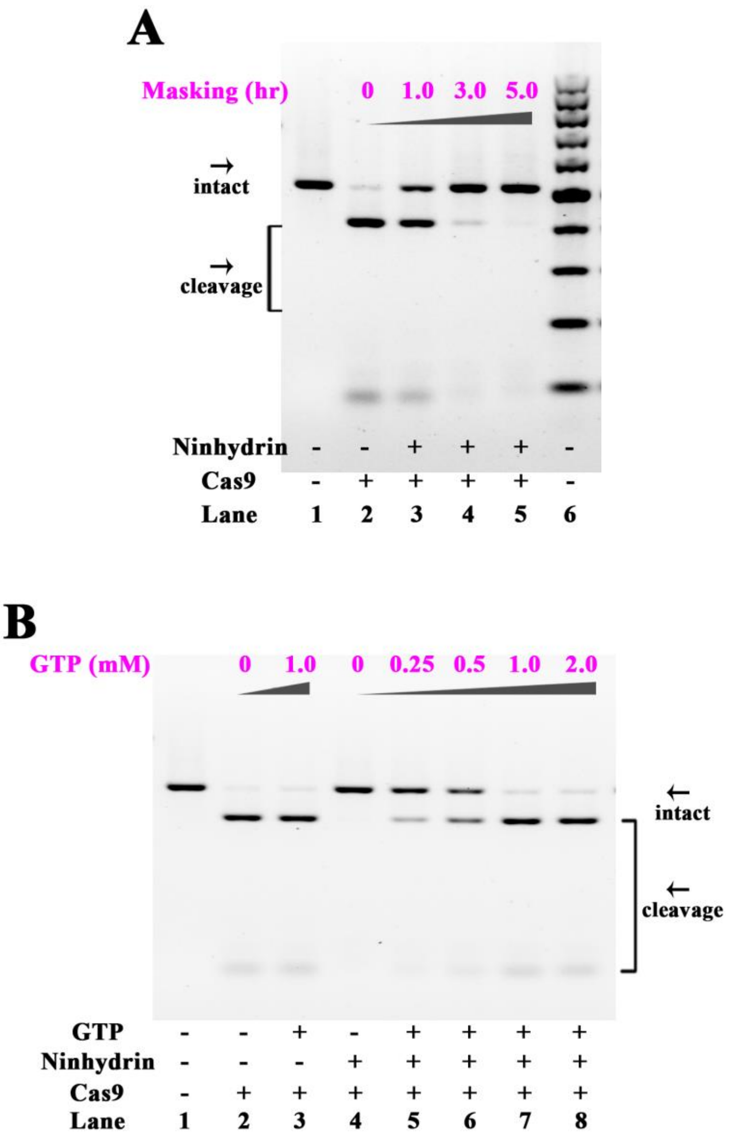

Figure S7. **Ninhydrin chemistry to controlling CRISPR/Cas9 system**. Reactions have been performed as described in the Experimental Section. All samples have been tested in three biological replicates. Image of representative data is shown here. The t-GFP2 DNA (500 bp) cut to shorter cleavage fragments (421 bp and 79 bp) are demonstrated. (**A**) The influence of ninhydrin masking on DNA cleavage. Lane 1: no Cas9 control; lane 2: original gGFP1; lanes 3 - 5: gGFP1 with different masking levels; lane 6: DNA markers. (**B**) The influence of GTP unmasking on DNA cleavage. The ninhydrin-masked gGFP1 (5 g/L ninhydrin, 5 hr) has been incubated with different concentrations of GTP for 5 min.

A

GAGGAGCTGTTCACCGGGGTGGTGCCCATCCTGGTCGAGCTGGACGGCGACGTAAA
CGGCCACAAGTTCAGCGTGTCCGGCGAGGGCGAGGGCGATGCCACCTACGGCAAGCT
GACCC**TGAAGTTCATCTGCAC**^**CACC**GGCAAGCTGCCCGTGCCCTGGCCCACCCTC
GTGACCACCCTGACCTACGGCGTGCAGTGCTTCAGCCGCTACCCCGACCACATGAAG
CAGCACGACTTCTTCAAGTCCGCCATGCCCGAAGGCTACGTCCAGGAGCGCACCATCT
TCTTCAAGGACGACGGCAACTACAAGACCCGCGCCGAGGTGAAGTTCGAGGGCGACA
CCCTGGTGAACCGCATCGAGCTGAAGGGCATCGACTTCAAGGAGGACGGCAACATCC
TGGGGCACAAGCTGGAGTACAACTACAACAGCCACAACGTCTATATCATGGCCGACA
AGCAGAAGAACGGCATCAAGGTGAACTTCAAGATCCGCCACAACATCGAGGACGGC
AGCGTGCAGCTCGCCGACCACTACCAGCAGAACACCCCCATCGGCGACGGCCCCGTG
CTGCTGCCCGACAACCACTACCTGAGCACCCAGTCCGCCCTGAGCAAAGACCCCAAC
GAGAAGCGCGATCACATGGTCCTGCTGGAGTTCGTGACCGCCGCCGGGATCACTCTC
G**GCATGGACGAGCTGTACAAG**

B

GACGTAAACGGCCACAAGTTCAGCGTGTCCGGCGAGGGCGAGGGCGATGCCACCTA
CGGCAAGCTGACCC**TGAAGTTCATCTGCAC**^**CACC**GGCAAGCTGCCCGTGCCCTGG
CCCACCCTCGTGACCACCCTGACCTACGGCGTGCAGTGCTTCAGCCGCTACCCCGACC
ACATGAAGCAGCACGACTTCTTCAAGTCCGCCATGCCCGAAGGCTACGTCCAGGAGC
GCACCATCTTCTTCAAGGACGACGGCAACTACAAGACCCGCGCCGAGGTGAAGTTCG
AGGGCGACACCCTGGTGAACCGCATCGAGCTGAAGGGCATCGACTTCAAGGAGGACG
GCAACATCCTGGGGCACAAGCTGGAGTACAACTACAACAGCCACAACGTCTATATCA
TGGCCGACAAGCAGAAGAACGGCATCAAGGTGAACTTCAAGATCCGCCACAACATCG
AGGACGGCAGCGTGCAGCTCGCCGAC**CACTACCAGCAGAACACCCC**

Figure S8. Schematic illustration of target sites of the gRNA in the GFP gene The purple underlines indicate the target loci of gRNA (gGFP2) and black caret shows the cleavage sites. Two GFP targets (t-GFP1 and t-GFP2) were amplified from pEGFP-C1 vector. (**A**) The demonstration of t-GFP1. (**B**) The demonstration of t-GFP2.

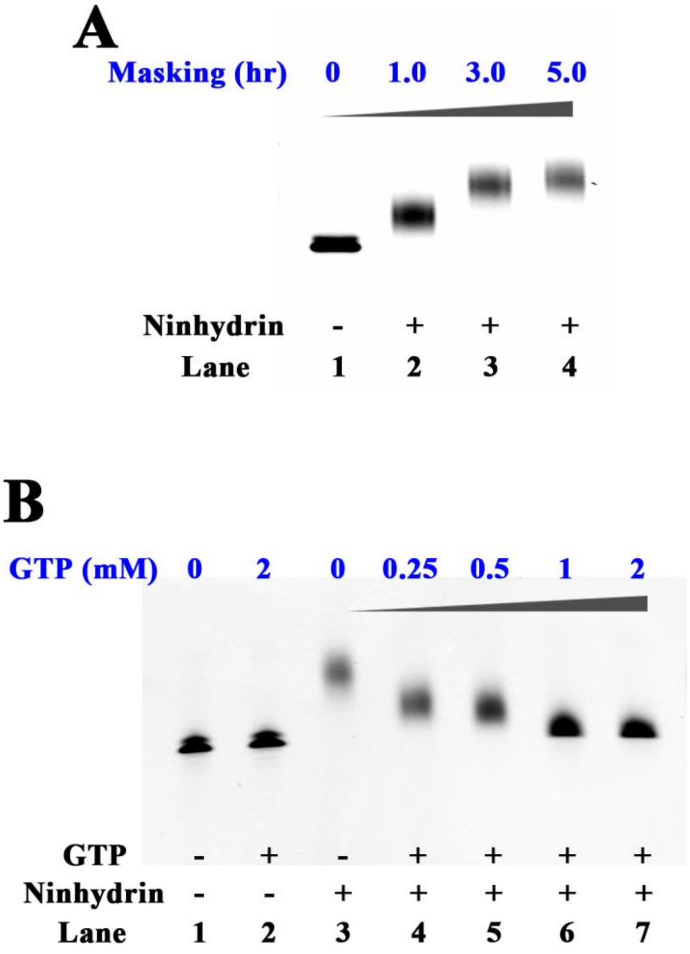

Figure S9. Ninhydrin chemistry to controlling CRISPR/Cas9 system. Reactions have been performed as described in the Experimental Section. All samples have been tested in three biological replicates. Image of representative data is shown here. (**A**) The ninhydrin masking of gRNA. The gRNA (gGFP2) has been incubated with 5 g/L ninhydrin for different periods. (**B**) The unmasking of ninhydrin-masked gGFP2. The ninhydrin-masked gGFP2 (5 g/L ninhydrin, 5 hr) has been incubated with different concentrations of GTP for 5 min.

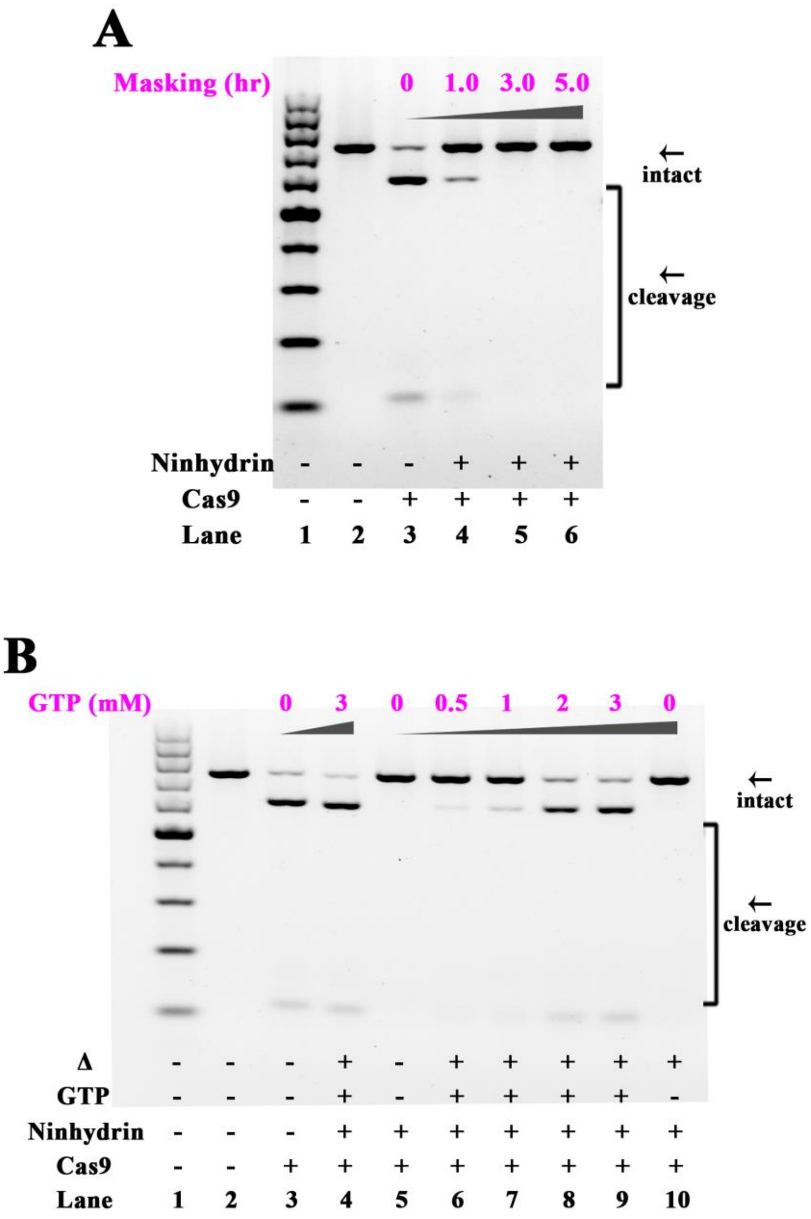

Figure S10. Ninhydrin chemistry to controlling CRISPR/Cas9 system. Reactions have been performed as described in the Experimental Section. All samples have been tested in three biological replicates. Image of representative data is shown here. The t-GFP1 DNA (702 bp) cut to shorter cleavage fragments (568 bp and 134 bp) are demonstrated. (**A**) The influence of ninhydrin masking on DNA cleavage. Lane 1: DNA markers; lane 2: no Cas9 control; lane 3: original gGFP2; lanes 4 - 6: gGFP2 with different masking levels. (**B**) The influence of GTP unmasking on DNA cleavage. The ninhydrin-masked gGFP2 (5 g/L ninhydrin, 5 hr) has been incubated with different concentrations of GTP for 5 min.

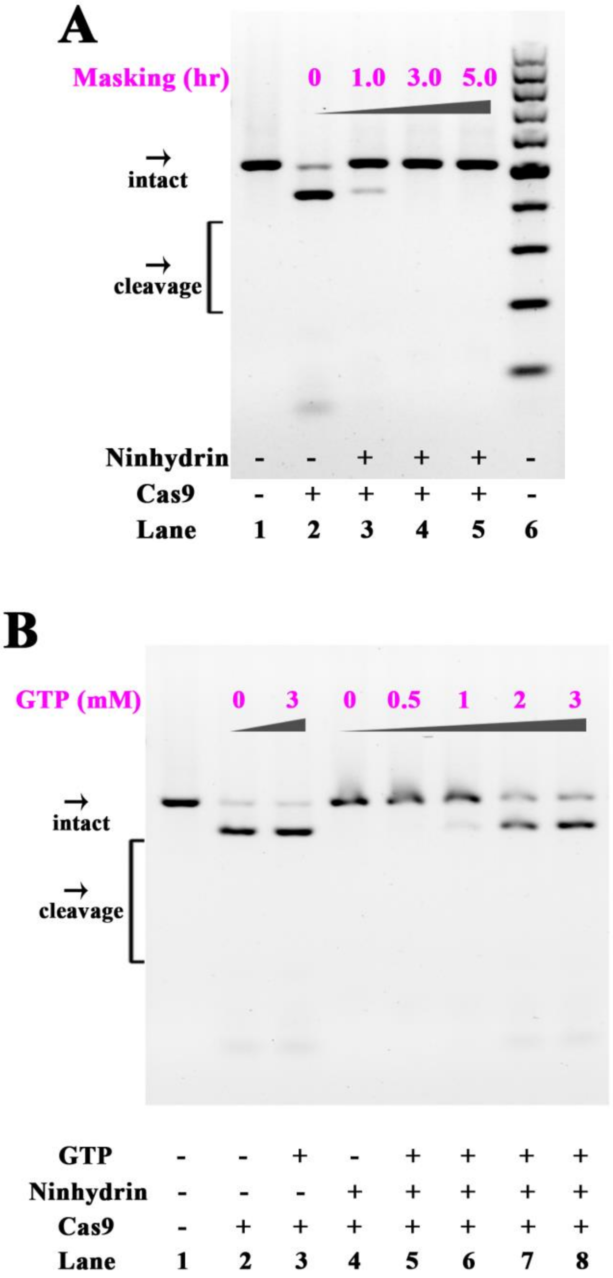

Figure S11. Ninhydrin chemistry to controlling CRISPR/Cas9 system. Reactions have been performed as described in the Experimental Section. All samples have been tested in three biological replicates. Image of representative data is shown here. The t-GFP1 DNA (500 bp) cut to shorter cleavage fragments (414 bp and 86 bp) are demonstrated. (**A**) The influence of ninhydrin masking on DNA cleavage. Lane 1: no Cas9 control; lane 2: original gGFP2; lanes 3 - 5: gGFP2 with different masking levels; lane 6: DNA markers. (**B**) The influence of GTP unmasking on DNA cleavage. The ninhydrin-masked gGFP2 (5 g/L ninhydrin, 5 hr) has been incubated with different concentrations of GTP for 5 min.

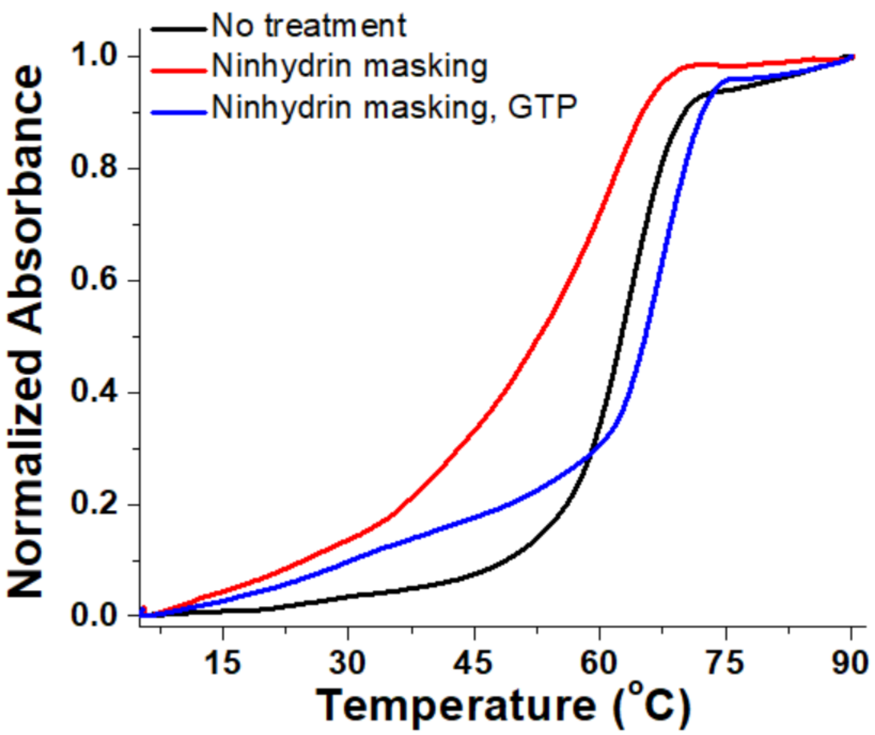

Figure S12. UV melting studies. Representative melting profiles of the crRNA/target RNA with different treatments were recorded in 10 mM Tris-HCl buffer (pH 6.8, 50 mM NaCl). Black line: no treatment control, 62.3 ± 0.2 °C; red line: the ninhydrin-masked crRNA1 (5 g/L ninhydrin, 5 hr), 53.2 ± 0.3 °C; blue line: the ninhydrin-masked crRNA1 after unmasking (2 mM GTP, 5 min), 65.4 ± 0.4 °C.
